# Using and improving the PHISICC paper-based tools in the health facility laboratories: Examples of Human Centered Design taking systems thinking into practice, in Côte d'Ivoire and Nigeria

**DOI:** 10.3389/fpubh.2022.916397

**Published:** 2022-09-15

**Authors:** Nnette Ekpenyong, Kathrin Heitz Tokpa, Ogonna Nwankwo, David O'Donnell, Damaris Rodriguez Franco, Salimata Berté, Simplice Amani Kouassi, Glory Eteng, Veronica Undelikwo, Christian Auer, Gouzan Bernard Guessan Bi, Angela Oyo-Ita, Xavier Bosch-Capblanch

**Affiliations:** ^1^Department of Community Medicine, University of Calabar Teaching Hospital, Calabar, Nigeria; ^2^Centre Suisse de Recherches Scientifiques en Côte d'Ivoire, Abidjan, Côte d'Ivoire; ^3^University of Basel, Basel, Switzerland; ^4^Swiss Centre for International Health, Swiss Tropical and Public Health Institute, Allschwil, Switzerland; ^5^Post Normal, Chicago, IL, United States; ^6^Sonder Collective, Madrid, Spain; ^7^Ecological Research Center, University of Nangui Abrogoua, Abidjan, Côte d'Ivoire; ^8^Ministry of Health and Public Hygiene, Directorate General of Health, Abidjan, Côte d'Ivoire; ^9^Department of Social Work, University of Calabar, Calabar, Nigeria; ^10^Department of Sociology, University of Calabar, Calabar, Nigeria

**Keywords:** health information system (HIS), quality of care, equity, Nigeria, Côte d'Ivoire, Human Centered Design, health workers, decision-making

## Abstract

**Background:**

Health workers in low- and middle-income countries are increasingly demanded to collect more and more data to report them to higher levels of the health information system (HIS), in detriment of useful data for clinical and public health decision-making, potentially compromising the quality of their health care provison. In order to support health workers' decision-making, we engaged with partners in Côte d'Ivoire, Mozambique and Nigeria in a research project to conceive, design, produce, implement and test paper-based health information tools: the PHISICC tools. Our aim was to understand the use of PHISICC tools by health workers and to improve them based on their feedback.

**Methods:**

The design Health Facility Laboratories (HF Labs) in Côte d'Ivoire and in Nigeria were set up after months of use of PHISICC tools. Activities were structured in three phases or ‘sprints' of co-creative research. We used a transdisciplinary approach, including anthropology and Human Centered Design (HCD), observations, shadowing, structured interviews and co-creation.

**Results:**

Health workers appreciated the standardization of the tools across different health care areas, with a common visual language that optimized use. Several design issues were raised, in terms of formats and contents. They strongly appreciated how the PHISICC registers guided their clinical decision-making and how it facilitated tallying and counting for monthly reporting. However, adherence to new procedures was not universal. The co-creation sessions resulted in modifications to the PHISICC tools of out-patient care and postnatal care.

**Discussion:**

Although health systems and systemic thinking allowed the teams to embrace complexity, it was the HCD approach that actually produced a shift in researchers' mind-set: from HIS as data management tools to HIS as quality of care instruments. HCD allowed navigating the complexity of health systems interventions due to its capacity to operate change: it not only allowed us to understand how the PHISICC tools were used but also how to further improve them. In the absence of (or even with) an analytical health systems framework, HCD approaches can work in real-life situations for the ideation, testing and implementation of interventions to improve health systems and health status outcomes.

## Background

Frontline health workers in low- and middle-income countries (LMIC) are increasingly asked by governments and donors to collect more and more data on their activities and to report them to higher levels of the system, compromising their dedication to health care service delivery and overloading them with routine health information systems (HIS) demands (1, 2). More recently, digital tools have come into play and been promoted by international organizations (3). While the potential gains brought by digital systems are enormous, they can also worsen this situation as they can easily cause data proliferation and redundancy (4). Furthermore, in many areas the infrastructure and services to support digital tools are infeasible at present and, in some cases, in the foreseeable future supporting the approach of hybrid systems (5). In order to support frontline health workers' decision-making, addressing these issues, we engaged with partners in Côte d'Ivoire, Mozambique and Nigeria in a research project to conceive, design, produce, implement and test innovative paper-based information tools: the “Paper-based Health Information Systems in Comprehensive Care” (PHISICC) research programme (from 2016 to 2021) (6).

PHISICC was conducted by a transdisciplinary team with team members from a range of disciplines including from public health and social sciences, to ministry decision-makers, frontline health workers, design researchers and graphic and interaction designers. The project synthesized the global evidence on health information systems; characterized the HIS in the three countries, focusing on opportunities for intervention in the HIS; and redesigned, using a co-creative Human Centered Design (HCD) approach, a suite of health information tools. The PHISICC tools covered most Primary Health Care services areas (i.e. antenatal care, deliveries, postnatal care, vaccinations, sick child, outpatients, tuberculosis, HIV and referral) and included the patient registers, tallies and the monthly reports. The tools were tested for their effectiveness on data quality and use and health outcomes, as well as health worker satisfaction in a cluster randomized controlled trial (RCT) in each of the three countries.

“Systems thinking is an approach to problem-solving that views problems as part of a wider dynamic system. It recognizes and prioritizes the understanding of linkages, relationships, interactions and interdependencies among the components of a system that give rise to the system's observed behavior” (7). While the practice of systems thinking is often focused on the comprehensive and insightful *mapping* of systems, the implicit or explicit goal of that mapping is to identify opportunities for *intervention* into that system (8). The question is, though, how to design interventions which effect change in health system performance and, eventually, in population health outcomes (9).

PHISICC followed the established HCD practice of engaging health workers early in the intervention design process as partners in the intervention's design rather than as passive informants whose role is to provide feedback on concepts developed by separate, “expert” designers (10). Holeman and Kane have described HCD as “frequently involving:

- meaningful and documented participation of people who will use new systems in their routine activities or otherwise be affected by them;
- supporting cooperative activity and augmenting people's skills, rather than using technology primarily for purposes of efficiency or managerial control; and
- concern for the whole person and their life experiences, reframing purely technical issues in relation to people's values and the broader human context of implementation” (11).

While evidence of the health outcome benefits of HCD are still emergent (12), this approach is increasingly being used in the field of public health. A growing body of evidence suggests that HCD affords significant opportunity to improve health outcomes (13) as it can “help the health community shift from prescribing solutions according to a *perception* of people's needs, to identifying solutions that actually meet their needs” (13). HCD brings a vitally important *tangibility and specificity* to the process of intervention design within the broader practice of systems thinking which, by its inherent comprehensiveness, tends toward *abstraction and generality*. If systems thinking helps us to “see the forest for the trees,” HCD helps us “see the trees for the forest”.

For the purposes of this report, we define HCD as follows: a research, design and problem-solving process in which knowledge about the topic of study is generated in dialogue with people directly involved in the topic and in which solutions to the identified challenges are created with the direct collaboration of those people most likely to benefit from them.

Concurrent with the PHISICC RCT, the PHISICC team continued to track the use, and improve the performance of the PHISICC intervention in a separate set of facilities, that we called the “Health Facility Laboratories” (HF Labs). The aims of the HF Labs were to deepen our understanding of the functionality of PHISICC tools, as well as to continue to improve the PHISICC tools in collaboration with health workers. We discuss the implications of HCD as a methodology in health systems thinking. This paper reports on the approaches, rationale and lessons learned from the HF Labs. Qualitative findings related to the trials outcomes are being reported together with the trials' quantitative findings in a forthcoming publication.

## Methods

The HF Labs were conducted at selected three health facilities in Côte d'Ivoire and five in Nigeria. These facilities were not included in the data collected for the RCT. All health facilities were drawn from the same study sites where the trial took place, but from those that were not selected as intervention or control health facilities for the trials. Although Mozambique was also a study site for PHISICC, the team there did carry out the RCT but without the HF Labs component, due to logistical constraints. The methods of the RCT trials are fully reported elsewhere (14).

**Figure 1:**
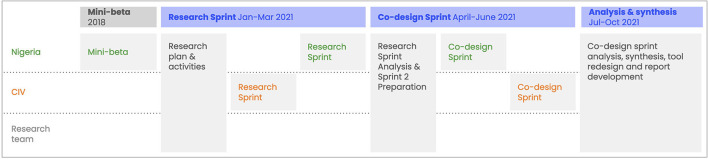
Health facility labs, overall approach and timeline.

The Health Facility Labs included three phases of co-creative research and design (Figure 1):

1. A phase where the PHISICC tools were piloted in Nigeria (we called it mini-beta) from June 2019 up to July 2019, preceding the start of the RCT, in which dialogues with health workers and observations of the tools' in-context use provided the insights to make final improvements to the tools before they were deployed in the trials;
2. At the very end of the RCT, we conducted a “Research Sprint” and assessed the perceptions and experiences of the health workers in the eight HF Labs facilities (three in Côte d'Ivoire and five in Nigeria) regarding the PHISICC tools;
3. And a series of “Co-design Sprints” in which health workers, researchers and designers discussed additional incremental improvements to the tools based on the health workers' experiences with them.

Selected health facilities were of primary health care level, in rural settings and often remote. They were: Abbe Begnini, Achiekoi, Elevi in Côte d'Ivoire (health district of Agboville) and Abachor, Echumoga, Imaje, Okuku and Woleche Agi in Nigeria, Cross River State (Yala Local Government Authority). The HF Labs sites followed the same programme as those sites enrolled in the trials; namely, training on the new tools and use of the new tools with patients for over a year, replacing the usual, regular HIS tools. After 12 months of using the tools in Côte d'Ivoire and 18 months in Nigeria, the HF Labs design and research team re-engaged with the health workers at these eight facilities to discuss their experiences with using the tools. The HF Labs took place between February 2021 and June 2021 in both countries. The research sessions (“Research Sprint”) and series of co-design sessions (“Co-design Sprints”) were purposively organized sequentially between the countries to allow integration of cross findings from the different county visits and to further integrate new findings from the last rounds of visits into any follow-up visit.

The interdisciplinary team working with health workers in the HF Labs included public health researchers and academics in Nigeria and Côte d'Ivoire as well as designers and researchers involved in the initial fieldwork that characterized opportunities for intervention and the visual design of the tools themselves. Cross-country collaboration had to be restricted to video conferencing due to the challenges with international travel resulting from COVID-19 pandemic restrictions.

### Mini-beta pilot

The “mini-beta” was a rapid but instructive testing of the tools in five health facilities in Nigeria, where health workers were trained and used the tool for 6 weeks. The goal was to make any final design improvements and to ensure that no obvious errors could have unintended consequences in the use of the tools. At the end, the interdisciplinary team worked with health workers who had used the tools on a daily basis to organize the feedback and implement a set of final improvements to the tools prior to the start of the RCTs. Notes were taken in a field diary during interviews and observations of consultations, tallying and reporting.

### Assessing the experiences of health workers

The research phase followed 12–18 months of use of the tools. After the trial period, health workers who had used the tools for an extended period of time became more informed about the tools' use than the designer team. Multidisciplinary research teams visited the health facilities, observed health care processes and management and collected data *in-situ* through notes and conversations with health workers. The repeated visits to observe the everyday workings of the rural health facilities (5 h in 5 days per health facility, approximately) created a productive rapport between health workers and the interdisciplinary team. A combination of observations (i.e., “shadowing”), conversations and structured interviews following interview guides were used to discuss the tools with health workers. A range of different types of health care areas (e.g., antenatal care and vaccinations) and administrative work, tallying, counting and preparing the monthly reports for the district were observed and discussed.

In Nigeria, on the first 2 days, the team divided into two groups and spent an average of 5 h in each health facility. During each of the visit, the health workers were shadowed as they carried out their day-to-day activities, conversations were also held on their use of the different tools and the challenges they had experienced, as well as in-depth interviews on the different aspects of the tools. On subsequent visits, a field editor was assigned to a health facility and the whole day was spent in the facility.

In Côte d'Ivoire, a total of three health facilities were visited by a team of five, consisting of two researchers, one representative of the Ministry of Health and two monitoring agents from the district. The research team spent an average of 5 h in each of the three health facilities during ten non-consecutive days.

Following the fieldwork, the team of researchers, both in and outside of the countries, developed research themes and findings and discussed possible redesigns or modifications to the tools based on health workers' feedback.

### Co-design: Modifying and improving tools following their use

Following the research phase, co-design sessions with health workers were conducted in each country. The objectives of the co-design sessions were:

1. To respond to requests for improvements from the health workers participating in the HF labs and using the PHISICC tools.
2. To collect direct feedback and input on these proposed additions to the suite of PHISICC tools.
3. To engage in a creative dialogue with health workers about the merits and areas of improvements for the PHISICC tools.
4. To catalog additional ideas and critiques from health workers based on their experience using the PHISICC tools.

In Nigeria, the team conducted two separate co-design sessions, for which six health workers from the previous research phase were recruited. The sessions lasted 5 h on average. In the first session, the team had a group discussion using an interview guide. In the second session, the team worked in three groups creating the sketched copy of the outpatient (OPD) register and tallies, and thereafter the research team met to merge and synthesize findings.

In Côte d'Ivoire the research team organized two co-creation sessions. The first session lasted 5 h and was conducted with all four members of the three health facilities that were involved in the HF Labs, and the second co-creation session lasted 3 h and was attended by four healthcare workers from four health facilities who had been part of the trial.

Each co-creation session followed the following pattern: all the PHISICC tools (i.e., the eight health care areas covered by PHISICC) were considered for possible improvements. This led to a focus on two health care areas: OPD and postnatal care (PNC) registers. Different features of each register were offered as separate pieces of paper. Each of the participating health workers created their own proposed design and explained the reasons to the group in a round of “presentations.” In this way, all health workers were able to ask questions and comment on the proposition and discuss problematic issues.

Consequently, during the analysis and synthesis phase (Figure 1), observations and findings were collaboratively organized into recurrent themes. Each different PHISICC tool and register was discussed from the perspective of health workers comments and in-situ observations of researchers. These paired critiques and observations revealed patterns and initial findings. The teams in Nigeria and Côte d'Ivoire compared findings and discussed implications for further improvements to the tools. Open questions and topics for further investigation were identified and explored through dialogue with health workers during the second round of site visits.

This process of pattern identification is consistent with the practice of human-centered and co-design. It follows the practice of combining different domains of knowledge to co-create new tools and approaches which embody different perspectives (15).

### Studying the incorporation of PHISICC tools at district level

Rural, typically small health facilities have no designated administrative staff as such. However, administrative personnel of the health system at the state level and district level were involved during the training of health care workers for the mini-beta and their inputs were considered. Additionally, in Côte d'Ivoire, five members of staff at the district were interviewed about their experiences with entering monthly reports from the PHISICC tools into the district online HIS reporting system. Whereas, the PHISICC registers were designed to be used in rural health facilities, the data they gathered *via* tally sheets were shared with the broader health system in monthly reports sent to the district and then national administrative levels. Based on Marcus' dictum developed in ethnographic research, to “follow the people,” we followed the health worker and the reporting tool up to the health district and assessed the use of the tool there (16, 17), including different levels of the health system into our analysis. This allowed us to gain insights into how the tool is used at district level and to appreciate the needs in a second context apart from the health facility.

## Results

Our transdisciplinary collaboration focused on health workers' perspectives and on their use of the tools, in a way that shifted the center of expertise about the PHISICC tools from the design and management teams to health workers themselves. As one health worker in Nigeria said: the “PHISICC tool has become part of me.” The repeated interactions between social scientists, designers, public health researchers, health workers and health managers, including monitoring and evaluation officers, led to the construction of a shared, practical knowledge in which multiple fields of practice were syncretised through the tangible modification of an intervention. We report our findings in four sections: (1) the PHISICC tools and health workers processes; (2) design issues in the PHISICC tools; (3) re-design of PHISICC tools; and (4) work at district level.

### PHISICC tools and health workers processes

The PHISICC design and management team expected that certain aspects of the PHISICC tools could affect health workers' behavior in data management and clinical care. In the domain of data management, a new approach to monthly reporting was designed. In the usual approach, at the end of each month health workers have to fill a monthly report covering all health care activities carried out during the month. To complete this report, health workers have to browse through the various registry books in order to count the items that are included in the report (e.g., number of pregnant women by age group, number of vaccinations of each type of vaccine). In the case of vaccinations, in most sites they use tally sheets as vaccination activities take place, which are summarized at the end of the month without needing to go back to the vaccination registry. In PHISICC health facilities, we implemented tallying mechanisms in all health care areas, with the intention to reduce for the health workers the workload of having to access all registries again at the end of the month.

In both countries, there was a generalized welcoming of the new tallying and reporting procedures. However, the adherence to this process of seamless tallying (i.e., tallying at the end of each clinical encounter) was not universal (see section below). These processes only changed behavior in a limited and inconsistent manner. Discussions with health workers in the HF Labs revealed that this deeply routinized end-of-month scrutiny of registers is difficult to shift and not necessarily considered problematic or seen as a cause of data quality issues.

In the health care domain, one of the key features of the tools was to provide health workers with visual clues to signal severity and hence support referral decisions. Health workers reported that they sometimes felt stuck between a rock and a hard place when complying with the demands of the health system and fulfilling the expectations of the local populations alike, while still needing to deliver high quality health care. During one of the co-creation workshops, health workers shared their dilemma to provide treatment when they actually have to refer cases, if they go by the book. Transportation costs are often high for families. Hence, they expect rural health workers to provide treatment. Health workers also fear for their reputation in the village as professionals, if they refer fever cases that are generally considered by communities as not dangerous, i.e., not needing hospital treatment. Furthermore, particularly when it is late at night or on holidays, patients risk not getting higher-level health care in the next urban place. In short, health workers feel sometimes obliged to treat cases that they should actually refer. In order to protect themselves from the anger of the community members, they may provide treatment whereas the recording of patient data (e.g., temperature values) is arbitrarily modified to justify local treatments without causing controversy, which challenges the use of the PHISICC tools. In other occasions, even if the “referral” alerts seemed to work, additional challenges may jeopardize the provision of care. A health worker reported, for example, that at the very beginning of the COVID-19 crisis, an ambulance to refer a patient with respiratory difficulties only reached the health facility hours later. Unfortunately, the patient died later in town. The population got extremely angry, besieged the health facility and threatened health workers. With the intervention of the police and negotiation of community leaders, the situation eventually calmed down.

### Design issues in PHISICC tools

Health workers appreciated the standardization of the tools across different health care areas. This made it possible to build common concepts across health care areas (e.g., the standard clinical pathway, from anamnesis to clinical examination, diagnosis and treatment; the importance of vital signs; signs of severity that may suggest referral). The PHISICC recording tools had three main different concepts: the clinical course, the lifeline and the tabular formats. The former consisted of distributing the main clinically relevant data items in an organized way across a page, taking into account the flow in the process of care throughout all the required visits. It was present in antenatal care, delivery, sick child, HIV, tuberculosis and referral. The lifetime design was used only for childhood vaccination. The tabular form, not radically different from the usual paper tools, was used in postnatal care and general consultation records. Arguably, the most favorite PHISICC tool among the health workers in Nigeria was the vaccination register:

“You only have to tick the boxes.” (Health worker, Nigeria)

“It is easy to locate a client in the register using the book and page number.” (Health worker, Nigeria)

“It is easy to carry around; it serves as a companion when going on home visit.” (Health worker, Nigeria)

The main challenge of the vaccination form was that, for the “lifetime” concept to work, it was paramount that the information of a given child coming for subsequent vaccines be recorded where the child was registered in the first instance and to have a good grasp on estimating ages based on date of birth, even if approximatively.

While the PHISICC's overall design concept, which prioritizes supporting health workers' decision-making alongside quality data collection, was highly valued by health workers, several features of the tools were revealed to be challenging in their daily use by health workers themselves. Health workers pointed at some data items that were missing and that needed to be incorporated. For example, there was no space to include new vaccines in the vaccination register. This issue came up when measles booster vaccine at 15 months was introduced into Nigerian National immunization schedule for children (18). They also mentioned the required additional space to accommodate antenatal care consultations, given the WHO recommendation to expand from six to eight consultations per pregnancy (19).

“Fully immunized should be 15 months. Extra spaces should be created for new vaccines that may be added to the immunization schedule.” (Health worker, Nigeria).

Health workers in Côte d'Ivoire requested to insert an extra space for the hour of the referral, in addition to the date. This is because, when they refer patients, it may take the family some time to bring up the money to actually start moving to the next town. In unfortunate cases, the patient dies and in case of a subsequent investigations, health workers would have the possibility to show proof of the hour the patient was referred.

There was actually a difficult choice between reserving space for changes and adaptations, which could be implemented even manually, and using the available space for clinical data considered relevant at the time of the ideation and production. The formal consistency of the design is essential to keep the visual language and its functionality. Hence, there were rather very limited alternatives to contemplate potential changes in health care recommendations in the future. One example of “manual” adaptation was from Côte d'Ivoire, where health workers used signs to mark HIV positive patients in a veiled/obfuscated manner, to ensure other patients will not be able to see the status of the previous patient during the consultation, when looking over the desk.

An issue that was present all along the design phase of the tools was the need to gather all information about the same client on the same page, particularly for those health care areas that require successive encounters for any client; i.e., antenatal care, postnatal care, vaccination, tuberculosis and HIV. While this is desirable from a clinical point of view, it entails a certain level of effort to search back in the register book every time a client shows up for any of those health care areas. This may be particularly challenging for health workers in large health facilities, with many clients and where information on current clients may span across several books. The advantage was that common data (e.g., name, contact details, basic biodata) does not need to be repeated at each consultation. This was highly valued by health workers:

“We only register the patient once at their first visit.” (Health worker, Côte d'Ivoire)

“There is no repetition of biodata when client comes for subsequent visit.” (Health worker, Nigeria)

“There was a link with the home-based card which made it easier to trace clients as they come for subsequent visits (vaccination register).” (Health worker, Nigeria)

“We note the register and page in the home-based record. This allows us to find the patient easily in the PHISICC book.” (Health worker, Côte d'Ivoire)

Besides the obvious gains, also referred by health workers, in time used for data recording, health workers identified other possibilities to further reduce the data recording efforts; e.g., using carbon copies:

Referral booklet “-should be made triplicate to reduce repeated writing of information and time wasting.” (Health worker, Nigeria)

Carbon copies were considered at the beginning of the design process but not implemented due to cost implications and potential issues with the quality of the copies.

### The redesign of PHISICC tools

The redesign of PHISICC tools took place based on feedback from health workers, which the team translated into designs through a process of “triangulation.” Decisions about design improvements resulted from the combination of health workers' practical experience, an anthropologist's interpretative take on the social and institutional interactions observed, public health experts' assessments of established clinical protocols, and a designer's ability to translate those combined perspectives into literal, tangible changes to the design of the tool. In this way, the (re)design of the intervention was not removed (in time and space) from field research and health workers themselves but directly integrated into the process of observation, analysis and critique.

The HF Labs research and co-creation sessions resulted in a series of important modifications to the PHISICC tools. The OPD and PNC registers were prioritized because these were the two health care areas where the implementation of the PHISICC design concept was limited and the tabular structure of the usual tools kept.

**Figure 2:**
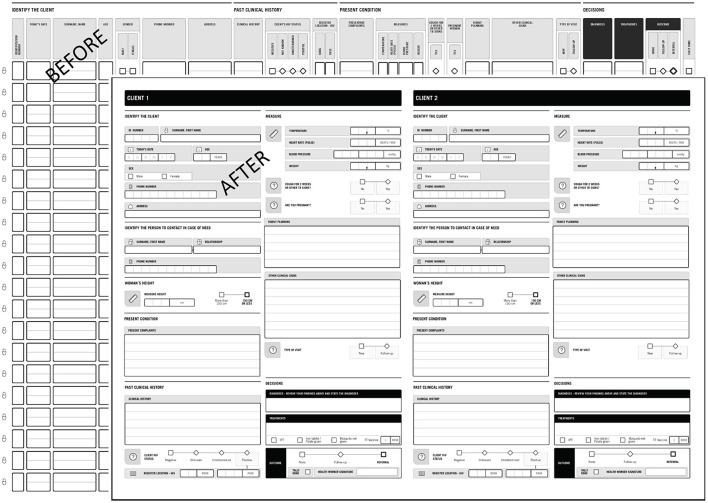
PHISICC outpatient register, before and after the series of co-design sessions.

**Figure 3:**
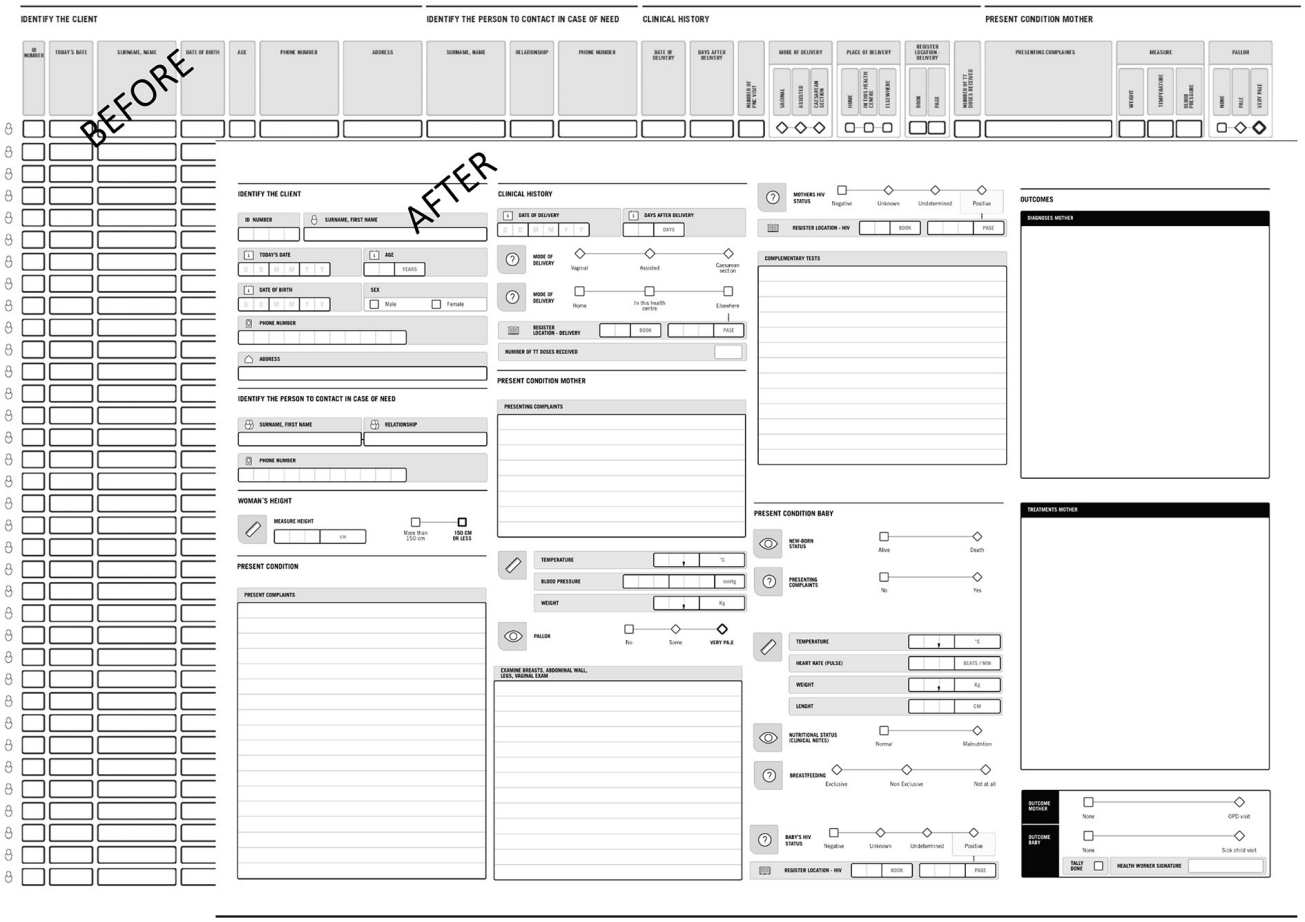
PHISICC postnatal care register, before and after the series of co-design sessions.

- The PHISICC OPD was modified to more closely resemble the design principles established in the other registers. The modified version provides more guidance for decision making, reorders the sequence of findings and arranged data collection to be more in keeping with the order of OPD consultations with patients. Consequently, the number of patients per page decreased due to the changes above (see Figure 2).
- The PHISICC PNC register was modified in order to contain only one client per page, providing health workers with more space to consider the patient presentation and make tool-guided decisions based on that presentation. The previous version contained 30 clients per page (see Figure 3).
- The monthly data tallies were designed as separate single sheets, for each health care area. They were redesigned so that they could be bound into books by health care area. The intent was to simplify the storage of the tools (i.e., the tallying/reporting sheet) and make sure the separate tally sheets, which health workers said were difficult to handle individually, do not get lost.
- The antenatal care (ANC) register kept the same structure and overall design but incorporated health workers' feedback by changing the orientation of “Date for next visit” field to the end of the visit, adding decimals spaces for “Weight”, adding the “Heart Rate” field, and adding a visual divider between systolic and diastolic values in the “Blood Pressure” item.

The re-designed OPD and PNC tools reduced the number of clients/patients per page to one. This has disadvantageous implications for retrieval and archiving of information because more paper may need to be used. However, we have also to consider that (a) we substantially reduced the overall size of the books to DIN-A3; (b) we reduced the number of registers (e.g., in Nigeria we canceled the general registration book at entrance, which provided no additional value to the whole information setup); and (c) regular books are often only partially filled in because of inconsistent data items, or because some pages are left blank to indicate sections within registers.

### At district level

The PHISICC tools, although focusing on primary health care health facilities, had to convey information to the district level through the monthly reports. Hence, both the mini-beta and the early stages of the trials required the involvement of actors at higher levels of the health system, the main issue being the inclusion of initially missing indicators into the PHISICC tallies and reporting tools. In particular, it was ensured that the indicators being reported at the facility level were aligned with the needs and expectations of the regional and national HIS. Although the project engaged the Ministry of Health at the highest levels from the start of the PHISICC project, there were communication challenges at the beginning of the implementation phase in Côte d'Ivoire, partially due to the COVID-19 situation. Whereas, the rural health facilities as well as the Ministry were well-informed, regional and district levels were not sufficiently associated at trial implementation. The supervisors of the health workers in Côte d'Ivoire lacked training on how to use the tools, as they had not been part of the training sessions. Furthermore, the format of the indicators of the new reporting tool did not perfectly match with the online reporting tool at district level. Within the district, health workers quickly delivered the missing indicators to the district by using a separate list or filing in national reporting forms that covered information that the tools initially did not. To improve the smoothness of the trial, a workshop was organized during which the region and districts were represented. This process allowed the project to include indicators of the national system into the PHISICC tools, making them fully compliant with the data requirements of the system.

In Nigeria, there was engagement of the Ministry of Health at the three tiers of government—Federal, State and Local Government Area from the start of the PHISICC project. However, at the beginning of the implementation of the project, there were challenges in the monthly reports. Similar to the situation in Côte d'Ivoire, the PHISICC summary sheet did not match the online reporting tool used at the district level. To overcome this problem, health workers at the facility were made to submit 2 monthly summary forms, the PHISICC form and the National Health Management Information System forms. This was seen by the health workers as double work for them. To overcome this challenge, extra support in terms of training in the PHISICC report was provided to the district monitoring and evaluation officer by the PHISICC team. Thereafter there was seamless entry of data captured from the PHISICC summary sheet to the state database.

## Discussion

### PHISICC in the context of health systems

The PHISICC paper-based system can be seen as a moderately or highly complex intervention (20): it has several components (i.e., recording, counting, reporting), several targets (i.e., data collection, clinical decision-making, follow-up), it embraces several system levels (i.e., primary health care and districts), with little flexibility in its implementation, requiring considerable skills for health workers using the information and basic skills for users of health services providing the information. HIS, as any other intervention affecting the health system, may impact on how health care is planned and delivered and may ultimately influence the health of the population. Yet, despite the existence of some examples of evidence-informed recommendations (21), health systems interventions lack the regulatory mechanisms of pharmaceutical interventions (22), that protect communities from systems harms. Our challenge from the outset of the PHISICC research programme was to incorporate professional (systems) design in the intervention ideation and development, within a complex research programme.

The WHO Health Systems framework considers “system-design” in the “leadership and governance” building block; however, there are no further explanations in the document (23). We could not really draw much from it. Our systems thinking was rather influenced by the sustained observations, conversations and collaborations with health care workers themselves, which triggered a relocation of systems components from what we initially conceived: emphasizing health care over data collection; the use of data over the collection of data; the decisions made with data over the quantity of data collected; and the concerns of rural and underserved health facilities over the concerns of managers charged with overseeing those facilities.

### HF labs contributions

The HF Labs were the space for those structured, productive collaborations between a transdisciplinary team, which combined domains of knowledge including public health, health information, anthropology, sociology, design strategy, and interaction and graphic design, with the practical experience and pragmatic knowledge of health workers. The HF Labs, focusing on qualitative and HCD methods, were not equipped to address intervention effectiveness questions (24). However, we value the evidence suggesting that PHISICC made a substantial qualitative improvement in the working lives of the health workers: they were appreciated and seen as support tools in the delivery of protocol-driven, quality health care.

The HF Labs experience has shown that it is possible to engage in sustained iterative improvements of a system intervention simultaneously with a rigorous trial of the effectiveness of that intervention. Complementing trials with qualitative evidence is becoming standard practice, particularly in health systems interventions and in systematic reviews (25) that eventually inform policies. By “setting aside” a small number of sites where the intervention was being used during the broader RCT, the HF Labs provided practical, qualitative assessments of the tools by health workers, produced explanatory evidence that deepened our insights on how the PHISICC intervention may work and created an opportunity to improve the intervention.

We believe that two main issues may explain the success of the HF Labs in both producing explanatory evidence and in providing clues for improvements, at the same time. On the one hand, the transdisciplinary approach made it possible to refocus the PHISICC intervention toward quality of care, taking into account socio-cultural attitudes and expectations as well as economic and geographical constraints of communities, putting people at the center of Health Policy and Systems Research (26). Secondly, the HCD approach provided a solid mechanism not only to understand how the PHISICC intervention could work but also to actually operate a tangible improvement of the intervention informed by the evidence collected: “Design is essentially a practical and pragmatic discipline that combines knowledge creation and knowledge use” (27). This set-up was quite unique in its format and in the mix of expertise involved. While the HF Labs were conceived in the context of research, we hypothesize that they can be valuable routine mechanisms to monitor and improve the usability of health systems interventions by health workers.

### Health systems thinking and health systems failures

Both in Côte d'Ivoire and in Nigeria, frontline health workers are requested by health programmes and external projects and donors to collect more data. Ministries of Health authorities were well-acquainted with this issue although with limited capacity to address it, likely due to the competing interests of many parties. They were, though, part of the PHISICC research team (6) and we would consider them to be enthusiastic with the prospects of considering simplification and user-friendliness in the routine data management procedures.

However, despite all the care taken to account for the health system setup and broad context, we also experienced “system failures,” some of which, we believe, were hardly possible to anticipate; for example, there were reports of external interferences from vertical programmes even in PHISICC intervention health facilities; or the fact that the very same data items that facilitated clinical care and patient treatments could be seen as a controlling mechanism in the context of clinical audit that brings penalisations, resulting in a situation that cannot be appropriately handled by the system (e.g., an aborted referral). These real-life situations may escape systems thinking considerations to the extent that its analytical capacity, and that of the underlying framework, remains limited.

Health systems challenges are indeed gigantic, particularly in LMIC (28), and have been in the research and development agenda, in one way or another, for years (29). Despite the consensus that systems thinking may have a role in health systems strengthening initiatives, there is limited evidence demonstrating how systems thinking has been practically applied to solve real-world health system challenges (30). It may be that health systems thinking is too specific to a particular health systems framework [e.g., in terms of “building blocks” (23)] and cannot negotiate, for example, a broader set of topics to inform health systems research syntheses (31); it may be that systems thinking tries to attain too large a range of concepts and tools that jeopardizes its consistent application, even in research settings (32); or it may be that systems thinking is not sufficiently developed to hold the multiple dimensions of health systems governance (33), delivery (34) and financial (35) arrangements and implementation strategies (36) together.

### The human centered design in practice

Shadowing is an established HCD technique used to both observe participants in the context in question and to enter into a dialogue with participants as they do the work. Although it could alter the behaviors of those observed, when done tactfully and intentionally, it creates a productive dialectic between activities and verbal reflections on those activities. The reflections' “proximity” to the activity in question allows both the participant and the researcher to investigate minute details of the activity, challenges, and work practices which may be glossed over or lost during recall or an interview conducted “not in-context”.

We hypothesize that HCD can address system failures through a pragmatic approach, using “design thinking” to bring solutions where problems cannot wait. HCD, put into practice by transdisciplinary teams in co-creative dialogue with actual people in the system, may be just the approach to produce interventions that can operate in real-life situations. The HCD method used in the PHISICC project placed frontline health workers and health facility patients at the very center of our research, analysis, and design (26). Health workers are often alone and feel fragile in the midst of a population that often considers them as outsiders to their communities; and any research that aspires to improve the quality of care has to embrace the human factor. We believe that we have shown a way of doing that.

This tangible, people-centered focus on interventions also shifted the team's own perspectives, from the more traditional system-wide, top-down analysis of health data, data collection processes and data quality to health workers themselves. Our co-creative, HCD approach focused the team's work at the very point in the system where healthcare happens *before* it becomes data *about* that care. In this way, HCD methods contribute to the practice of systems theory in that they provide a replicable method for translating the multi-perspective insights inherent in the mapping of complex systems like health information systems into tangible, material interventions that incorporate the inputs of participants in, and observers of that same system.

### Research in remote areas

We would like to briefly mention the challenges of carrying out research in remote rural areas, including the availability of staff for training (already in shortage at their workplaces), staff turn-over, transport and communication means, living conditions, weather and geographical barriers. There does not seem to be a lot of evidence on the challenges of research in remote areas and on strategies to cope with them (37). This was only worsened by the COVID-19 pandemic because during this period the health workers were asked to focus on COVID-19 vaccination, leading to general disruptions in the use of routine health services and in the provision of healthcare during the period of the lock-down. Exchanges between research teams from Côte d'Ivoire and Nigeria were also affected by these restrictions. Only the commitment of the team to contribute to improving the lives of underserved communities could overcome those gigantic obstacles.

## Conclusion

HF Labs may serve as a model for how transdisciplinary design research, centered on the perspectives of health workers, can lead to the creation of a health system intervention, which, once produced and used for an extended period of time, can be further evaluated and improved, within a wider research programme. HCD can operationalise health systems thinking into health systems interventions operating in real-life situations, even in the absence of a fully developed and consistent health systems framework.

While we value and share the recently issued recommendations on health systems thinking (30), we would advocate for considering HCD approaches for the ideation, testing and implementation of interventions to improve health systems and health status outcomes.

## Data availability statement

The raw data supporting the conclusions of this article will be made available by the authors, without undue reservation.

## Ethics statement

The studies involving human participants were reviewed and approved by In Côte d'Ivoire: Comité National Ethique des Sciences de la Vie et de la Santé – CNESVS; in Nigeria: Government of Cross River State of Nigeria, Ministry of Health, Calabar Health Research Ethics Committee. Written informed consent for participation was not required for this study in accordance with the national legislation and the institutional requirements.

## Author contributions

XBC, DR, and DO were instrumental in the development of the concept and design of the study. NE, KH, ON, SB, SA, GE, and VU interacted with the health workers and other stakeholders in Côte d'Ivoire and Nigeria, respectively and gathered the data in the field. DO and DR guided the fieldwork remotely. XBC coordinated research teams, commented and provided substantial inputs to the research protocol, report of findings and manuscript. CA commented and provided substantial inputs to the research protocol, report of findings and manuscript. GBG supported the field work in Côte d'Ivore and AOI in Nigeria; both significantly contributed to the manuscript. All authors substantially contributed to conception and/or development of the study and the manuscript.

## Funding

The study was funded by the Bill & Melinda Gates Foundation. Grant number INV-010193/OPP1135947. BMGF funded the whole PHISICC research project.

## Conflict of interest

The authors declare that the research was conducted in the absence of any commercial or financial relationships that could be construed as a potential conflict of interest.

## Publisher's note

All claims expressed in this article are solely those of the authors and do not necessarily represent those of their affiliated organizations, or those of the publisher, the editors and the reviewers. Any product that may be evaluated in this article, or claim that may be made by its manufacturer, is not guaranteed or endorsed by the publisher.
